# Structural, Optical, and Thermal Properties of PVA/SrTiO_3_/CNT Polymer Nanocomposites

**DOI:** 10.3390/polym16101392

**Published:** 2024-05-14

**Authors:** Alhulw H. Alshammari

**Affiliations:** Physics Department, College of Science, Jouf University, Sakaka P.O. Box 2014, Saudi Arabia; ahalshammari@ju.edu.sa

**Keywords:** PVA polymer, SrTiO_3_/CNT nanofillers, thermal and optical characteristics

## Abstract

Successful preparation of PVA/SrTiO_3_/CNT polymer nanocomposite films was accomplished via the solution casting method. The structural, optical, and thermal properties of the films were tested by XRD, SEM, FTIR, TGA, and UV-visible spectroscopy. Inclusion of the SrTiO_3_/CNT nanofillers with a maximum of 1 wt% drastically improved the optical and thermal properties of PVA films. SrTiO_3_ has a cubic crystal structure, and its average crystal size was found to be 28.75 nm. SEM images showed uniform distribution in the sample with 0.3 wt% of SrTiO_3_/CNTs in the PVA film, while some agglomerations appeared in the samples of higher SrTiO_3_/CNT content, i.e., at 0.7 and 1.0 wt%, in the PVA polymer films. The inclusion of SrTiO_3_/CNTs improved the thermal stability of PVA polymer films. The direct and indirect optical band gaps of the PVA films decreased when increasing the mass of the SrTiO_3_/CNTs, while the single-oscillator energy (*E*_0_) and dispersion energy (*E_d_*) increased. The films’ refractive indices were gradually increased upon increasing the nanofillers’ weight. In addition, improvements in the optical susceptibility and nonlinear refractive indices’ values were also obtained. These films are qualified for optoelectronic applications due to their distinct optical and thermal properties.

## 1. Introduction

Polymers have garnered significant attention from researchers owing to their unique physical and chemical properties, coupled with their affordability, easy fabrication, stability across intermediate temperatures, transparency to visible and infrared wavelengths, and more [1,2,3]. Certain polymers like polyvinyl chloride (PVC) and polyvinyl alcohol (PVA) exhibit high transparency, enabling them to transmit a wide range of wavelengths, making them advantageous in various applications. PVA polymers are known for their ease of fabrication, high hydrophilicity, non-toxicity, availability, and dielectric strength, as well as their distinct optical, physical, and chemical properties [4,5,6]. Hence, PVA polymer holds promise for various applications due to the aforementioned features. Moreover, the properties of these polymers can be further enhanced through doping with suitable nanoparticles or nanofillers [7]. The properties of dopants, including their small size, optimal shape, and expansive surface area, serve as key factors that bolster the properties of the polymer [6].

Various studies have been conducted on polymer nanocomposites; however, the optical properties of PVA polymer have not yet been deeply investigated. The optical and structural properties of PVA were investigated when incorporating a low percentage of graphene oxide (GO) up to 1 wt%. The inclusion of GO into the PVA films reduced their direct and indirect optical band gaps [6]. Several studies have investigated the impact of GO on the properties of PVA polymer [8,9,10].

The structural, dielectric, and optical characteristics of PVA polymer were investigated upon incorporation of Ag-BaTiO_3_ nanofillers. The resulting PVA-Ag-BaTiO_3_ films exhibited favorable dielectric and optical characteristics, suggesting their suitability for electric and optoelectronic applications [11]. The structural and optical properties of PVA when doped with PANI/Ag nanoparticles have been studied, and its optical properties have a direct dependency on the nanoparticles’ mass [12].

Strontium titanate (SrTiO_3_) has been employed as a dopant for polymer films due to its distinct characteristics, i.e., a high dielectric constant, low dielectric loss, and minimal leakage current. With a relatively wide band gap energy of approximately 3.2 eV and a cubic perovskite structure at room temperature, SrTiO_3_ is anticipated to improve the thermal and optical performance of PVA polymer films when used as a dopant [3,13,14]. Hence, previously the thermal and dielectric properties of undoped PVA and doped PVA with SrTiO_3_ were examined. Despite the relatively high percentage of SrTiO_3_, ranging from 5 to 15 wt%, the PVA/SrTiO_3_ nanocomposites exhibited favorable performance [15].

On other hand, CNTs are expected to cause light scattering within PVA films, thus increasing the path length of photons, particularly in the UV–vis region. CNTs are sheets of graphene formed into tubes which are widely used for structural enhancements. CNTs are hundred-folds stronger than steel [16,17]. The inclusion of SrTiO_3_ into PVA can cause photon scattering. The presence of SrTiO_3_/CNTs in a polymer film is also expected to enhance the thermal stability of the polymer. A recent study has shown a positive impact on the optical properties of PVDF polymer when doped with SrTiO_3_/CNTs [17]. Therefore, using SrTiO_3_/CNTs as dopants in PVA films has a high potential to improve their optical and thermal stability. The influence of SrTiO_3_/CNTs on PVA films has not yet been investigated. Therefore, this work studies the impact of SrTiO_3_/CNT mixtures as dopants on the structural, thermal, and optical properties of PVA polymer films. The PVA polymer films doped with (0.0, 0.3, 0.7, and 1.0 wt%) SrTiO_3_/CNTs were synthesized via the solution casting method. The structural, optical, and thermal properties of these films were measured. Therein, various characterization techniques, i.e., XRD, SEM, TGA, and UV-visible spectroscopy, were utilized. The thermal and optical performance of PVA films were improved upon the inclusion of SrTiO_3_/CNTs. The distinct properties of PVA/SrTiO_3_/CNT polymer nanocomposite films indicate that the films have high potential to be included in optoelectronic applications.

## 2. Experimental

### 2.1. Materials

Polyvinyl alcohol (PVA), carbon nanotubes (CNTs), and strontium titanate (SrTiO_3_) nanopowder were purchased from Nanografi locating in Ankara, Turkey. Ethanol absolute was purchased from Sigma-Aldrich, Burlington, MA, USA.

### 2.2. Preparation Method and Characterization

A mixture of 0.2 g CNTs and 0.8 g SrTiO_3_ nanopowder was dissolved into 50 mL of ethanol for an hour. The mixture was exposed to ultrasonic waves for an hour and then dried inside an electric oven under 50 °C. Finally, the leftover product was finely ground. The solution casting technique was utilized to process the polymer films. As such, 1 g of PVA was added to 80 mL of deionized water under magnetic stirring for an hour at ambient temperature to be dissolved. Different contents of SrTiO_3_/CNTs, i.e., 0.003, 0.007, and 0.01 g, were added to the PVA solution samples and stirred regularly for an hour. The resulting solutions, each with a volume of ~80 mL, were poured into glass petri dishes and left to dry in an oven under 80 °C for 48 h. Then, the polymer films were peeled off and taken for characterization. The thickness and dimensions of these films were almost the same, where the average of the thickness was nearly 112 µm. A Shimadzu diffractometer (Kyoto, Japan) with model of 7000 and λ of CuK_α_ radiation = 1.54056 Å scanning in the range of 2θ = 10–80° was used to show the XRD patterns of the polymer films that consisted of the pure PVA and mixed PVA with 0.3, 0.7, and 1 wt% SrTiO_3_/CNTs. A Shimadzu spectrometer (FTIR–Tracer 100, Kyoto, Japan) scanning wavenumbers from 399 to 4000 cm^−1^ was utilized to record ATR spectra. A Thermo Fisher Quattro ESEM (Thermo Fisher Scientific, Waltham, MA, USA) was also used to produce FESEM micrographs of our samples. This advanced technique was crucial as a source of morphology and microstructure information about the polymer films. The mass loss of the polymer nanocomposite films was examined as function of temperature in the range of 30–600 °C by a Shimadzu TGA-51 Thermogravimetric Analyzer (Kyoto, Japan). A Cary 60 UV–vis spectrophotometer, Agilent (Santa Clara, CA, USA) at a wavelength scan 190–1000 nm was used to provide optical measurements.

## 3. Result and Discussion

### 3.1. Structural Characterization

**Figure 1:**
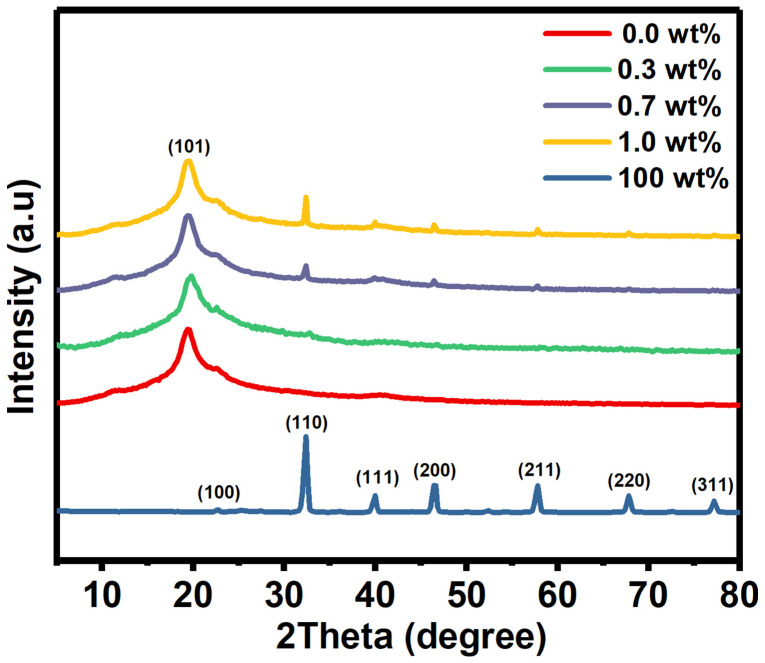
The XRD diffraction peaks of pure PVA and doped PVA with SrTiO_3_/CNTs.

XRD as a well-known identification technique, was used to obtain information about the crystal structure of the films. Here, the XRD patterns of pure PVA film, SrTiO_3_/CNTs, and doped PVA with 0.3, 0.7, and 1 wt% SrTiO_3_/CNTs are shown in Figure 1. A broad peak centered at 2θ = 19.4° was observed, resulting from the (101) plane of PVA in its monoclinic crystal structure [18]. The small peak appearing at 2θ = 40.6° indicates the semi-crystallinity structure of the PVA film [19,20]. The molecular strong interaction by intermolecular hydrogen bonding formed the nature of the PVA’s crystallinity [20].

The SrTiO_3_/CNT film has various peaks located at 2θ = 22.66°, 32.3°, 39.95°, 46.5°, 57.77°, 67.86°, and 77.25° which are assigned to the (100), (110), (111), (200), (211), (220), and (310) planes [3]. It is obvious that when increasing the SrTiO_3_/CNT content from 0.3 to 1 wt% in the PVA polymer films, the intensity at 2θ = 32.3° gradually increases as expected, since that peak is related to the (110) plane of SrTiO_3_. Here and based on XRD patterns, the SrTiO_3_ has a cubic crystal structure, as reported in JCPDS card (35–0734) [21].

The average crystal size of the nanoparticles can be calculated by the Debye–Scherrer method (see Equation (1)).

(1) $$D=\frac{\;0.9\lambda }{\beta \;\cos\;\theta }$$

D is the average crystal size, while λ and β are the wavelength of the X-ray and full width at half maximum, respectively. The SrTiO_3_ crystal size is an average of 28.75 nm.

**Figure 2:**
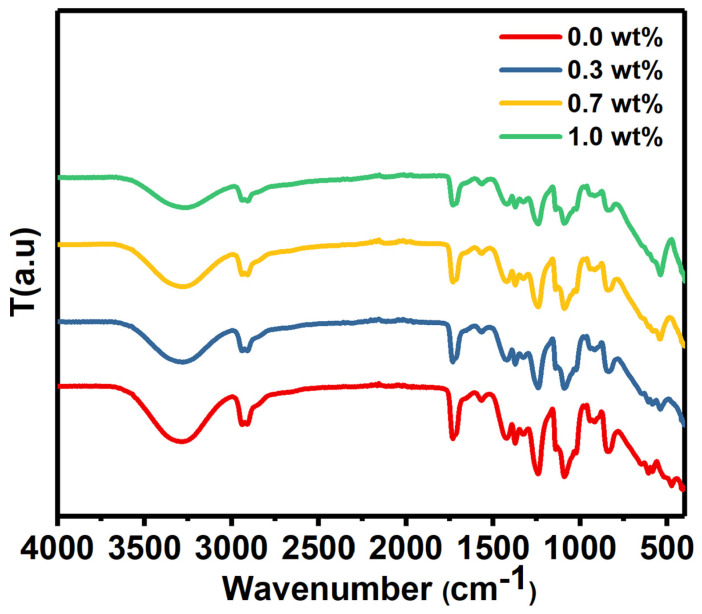
The FTIR spectrum of pure PVA and PVA doped with SrTiO_3_/CNTs.

The FTIR spectrum of PVA/SrTiO_3_/CNTs was measured from 400 cm^−1^ to 4000 cm^−1^_,_ as depicted in Figure 2.

The FTIR spectrum had a broad absorption band centered at nearly 3270 cm^−1^ for pure PVA film and doped PVA with different masses of SrTiO_3_/CNTs. These bands originated from –OH stretching vibration [22]. C–H stretching and out-of-plane C–H stretching caused the peaks located around 2920 and 1426 cm^−1^, respectively [23]. C–O stretching vibration caused the band at 1090 cm^−1^ [24].

**Figure 3:**
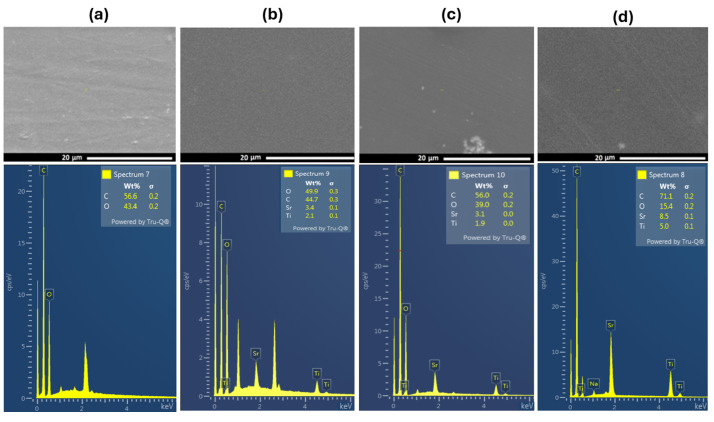
SEM scans of (**a**) pure PVA film and (**b**–**d**) PVA films doped with SrTiO_3_/CNTs.

The scanning electron micrograph images of PVA and PVA doped with SrTiO_3_/CNTs are shown in Figure 3. A SEM image of pure PVA film is shown in Figure 3a. The inclusion of a small content of SrTiO_3_/CNTs, i.e., 0.3 wt%, showed uniform distribution in the PVA polymer film, as depicted in Figure 3b. However, when the dopants’ content increased to 0.7 and 1 wt%, some agglomerations were formed, as shown in the bottom of the SEM images in Figure 3c,d. The formation of agglomerations can be attributed to the dopants and the conditions of the polymer film preparation [18]. The increased concentration of nanoparticles could prevent their uniform dispersion and interfacial interaction between polymer chains and nanoparticles [3,18,25]. The crosslinking between PVA and SrTiO_3_/CNTs is assigned to either strong hydrogen bonds or the increased viscosity of the polymer nanocomposites during the preparation process, and such phenomena have been previously reported in [3,26,27]. Figure 3a–d shows the EDS analysis of the included elements in PVA films incorporating 0.3, 0.7, and 1 wt% of SrTiO_3_/CNT samples, respectively. The EDX analysis evidences the increment of the SrTiO_3_ ratios in the prepared PVA polymer films. The corresponding elemental distribution mapping is illustrated in Figure S1 (ESI).

### 3.2. Thermal Stability

**Figure 4:**
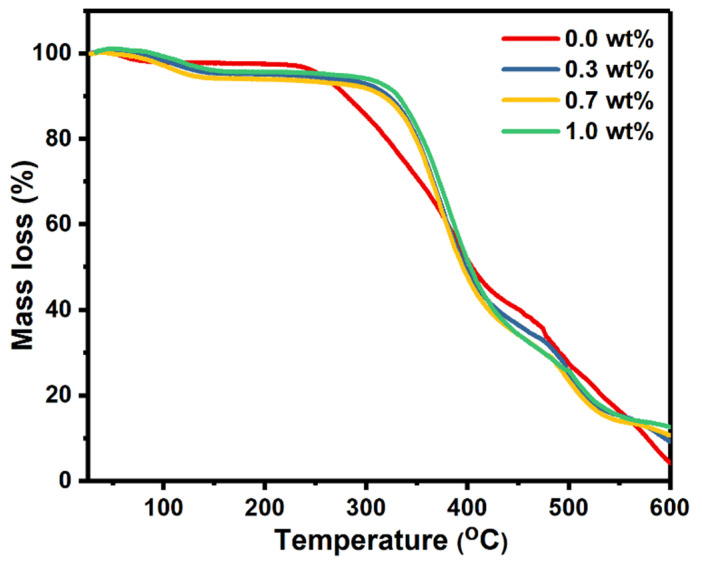
TGA scans of pure PVA films and PVA doped with SrTiO_3_/CNTs.

TGA analysis was used to investigate the thermal stability of the polymer nanocomposites under different temperatures. The mass losses of pure PVA and PVA doped with SrTiO_3_/CNT samples were plotted against the temperature, as shown in Figure 4.

It is obvious that there were three decomposition stages for all the polymer films. The first small degradation stage occurred in the range of 80–145 °C and is assigned to the evaporation of the solvent. For the pure PVA film, the second degradation stage occurred at 235–417 °C, while the third degradation stage began at 475 °C and continued up to the end of the scale at 600 °C. The doped PVA films with SrTiO_3_/CNTs also had two degradation stages at 310–424 °C and 500–550 °C. Incorporating the SrTiO_3_/CNTs into the PVA delayed the thermal degradation, since the second degradation stage of pure PVA began at 235 °C while the doped PVA remained stable up to 310 °C. It is noticeable that small additions of SrTiO_3_/CNTs with a maximum of 1 wt% kept the PVA films stable for 75 °C more than pure PVA. Therefore, it is obvious the thermal stability of the PVA films was clearly improved when incorporating SrTiO_3_/CNTs.

### 3.3. Optical Measurements

**Figure 5:**
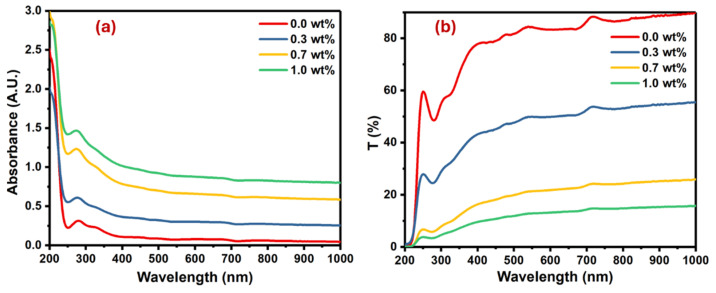
Plots for the PVA films doped with SrTiO_3_/CNTs: (**a**) absorbance versus wavelength and (**b**) transmittance versus wavelength.

The optical properties of pure PVA and PVA doped with different masses of SrTiO_3_/CNTs were investigated, and the results of their optical absorbance and transmittance spectra are shown in Figure 5.

The pure PVA film had an absorption band at 280 nm because of the π→π* interband electronic transitions [28,29]. Upon the addition of SrTiO_3_/CNTs into the PVA film, the absorption band slightly blue shifted due to band gap widening, as shown in Figure 5a. The magnitude of the blue shift intensified with higher concentrations of SrTiO_3_/CNTs, peaking at 6 nm when 1 wt% SrTiO_3_/CNTs was present. This significant increase suggests the successful preparation of polymer nanocomposites. The increase in the blue shift with higher concentrations of SrTiO_3_/CNTs can be attributed to the interactions between the nanoparticles and the polymer. As the concentration of SrTiO_3_/CNTs increases, there is a greater incorporation of these nanoparticles into the polymer matrix. This incorporation alters the optical properties of the nanocomposite material, leading to the absorption or emission wavelengths shifting towards the blue region of the spectrum. The peak shift reaching its maximum at 1 wt% SrTiO_3_/CNTs indicates optimal nanoparticle dispersion and interaction within the polymer matrix, reflecting the successful preparation of the nanocomposites. Moreover, as the concentration of SrTiO_3_/CNTs increased, it caused a band defect formation in the PVA film [30]. Figure 5b shows the transmittance of undoped PVA as well as doped PVA with SrTiO_3_/CNTs.

**Figure 6:**
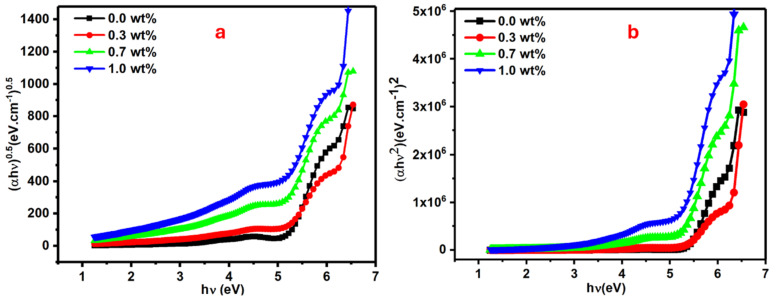
Plots for the PVA films doped with SrTiO_3_/CNTs: (**a**) (∝*hυ*)^0.5^ vs. *hυ* and (**b**) (∝*hυ*)^2^ vs. *hυ* for the PVA/SrTiO_3_/CNT films.

It is obvious the transmittance decreased with the increasing mass of SrTiO_3_/CNTs. This observation is typical, since the inclusion and dispersion of nanoparticles in the polymer films results in the scattering of incident photons. The optical band gap in the polymer film is usually obtained by the Tauc equation (see Equation (2)) [31,32]:

(2) $$\alpha h\upsilon =B{\left(hv-E_{g}\right)}^{n}$$

where *α* is a coefficient of absorption and *B* is a constant, while *hυ* indicates incident photon energy. The *n* value will be 0.5 in the case of direct allowed transitions and 2 in case of indirect allowed transitions. The direct and indirect band gap values can be estimated from the interception of the extended straight line of the curve to the zero absorption axis in Figure 6a,b.

The direct optical band gap for pure and doped PVA with 0.3, 0.7, and 1 wt% SrTiO_3_/CNTs gradually decreased to 5.06, 4.86, 4.76, and 4.50 eV, respectively. The tiny mass of the dopant in the range of 0.3–1 wt% caused drastic changes in the value of the direct band gap. The indirect optical band gap for pure and doped PVA with 0.3, 0.7 and 1 wt% SrTiO_3_/CNTs gradually decreased to 5.31, 5.10, 5.08, and 4.92 eV, respectively.

E_dir_ and E_ind_ values as listed in Table 1 are decreased with the increments of SrTiO_3_/CNTs. The substantial shift in the energy band gap of PVA, ranging from 5.06 to 4.50 eV, suggests that SrTiO_3_/CNT nanoparticles induce modifications in the electronic structure of the PVA matrix. This alteration is attributed to the localized electronic states formed by the incorporated SrTiO_3_/CNT nanoparticles within the optical band gap of PVA, functioning as trapping and recombination centers. Consequently, the observed change in the optical band gap occurs. Additionally, the decrease in the optical band gap may be attributed to an increase in the degree of disorder in the samples, resulting from changes in the polymer structure [33,34,35,36,37].

Investigating the refractive index of the polymer nanocomposite films is meaningful for determining their suitable applications. The films with the advantage of a high refractive index and electrical performance are qualified for optoelectronic applications. Some factors, i.e., molecular structure, nanofillers, film thickness, etc., can influence a film’s refractive index [38].

**Figure 7:**
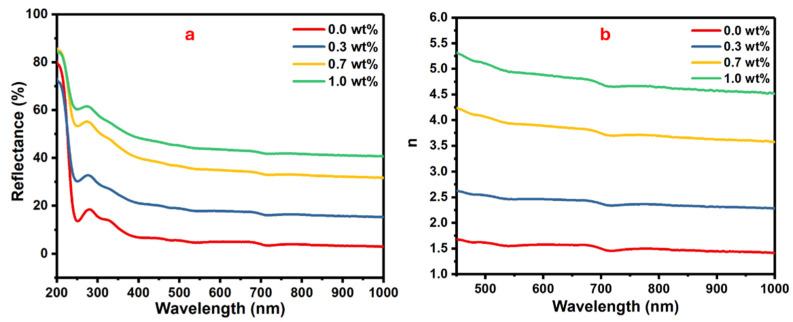
The (**a**) reflectance vs. wavelength and (**b**) the refractive index vs. wavelength for the PVA/SrTiO_3_/CNT polymer films.

The reflectance and refractive index as function of wavelength are plotted in Figure 7. Reflectance gives information about the amount of reflected light from a surface with respect to incident light.

The changes in the reflectance against the wavelength for the samples of PVA doped with different masses of SrTiO_3_/CNTs are shown in Figure 7a. Equation (3) shows the relationship between reflectance (*R*) and refractive index (*n*) [39,40].

(3) $$n=\left(\frac{1+R}{1-R}\right)+\sqrt{\begin{array}{c} \frac{4R}{{\left(1-R\right)}^{2}}-k^{2} \end{array}},$$

where the extinction coefficient is represented by (*k* =αλ/4π). The refractive indices are gradually increased with the increasing mass of SrTiO_3_/CNTs, as displayed in Figure 6b. The enhancement of the refractive index is attributed to the formation of a large cluster from the gathered SrTiO_3_/CNT nanoparticles [41]. The polymer films with a high high-refractive-index are qualified for optical device applications, i.e., optical coatings, anti-reflection screens, etc.

The material dispersion parameters, known as oscillator energy, dispersion energy, and transition moments, can be evaluated from refractive index dispersion as in Equation (4), which includes single-oscillator energy (*E*_0_) and dispersion energy (*E_d_*) [42].

(4) $${\left(n^{2}-1\right)}^{-1}=\frac{E_{0}}{E_{d}}-\frac{1}{E_{0}E_{d}}\;{\left(h\upsilon \right)}^{2}$$

**Figure 8:**
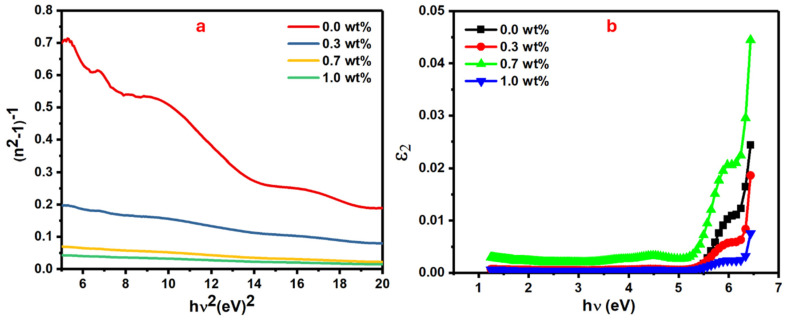
CNT (**a**) (n^2^ − 1)^−1^ versus ${\left(h\upsilon \right)}^{2}$, and (**b**) ε_2_ versus *h*υ.

Figure 8a shows the plot of the (*n*^2^ − 1)^−1^ vs. (*hυ*)^2^ where *E*_0_ and *E_d_* values are obtained from its slope and intercept. The *E_d_* values directly increase from 4.69 to 92.57 eV when the mass of SrTiO_3_/CNTs increases from 0 to 1 wt%, while the *E*_0_ values show an incremental trend from 4.48 eV to 4.98, as listed in Table 2.

The enhanced intermolecular interactions, which resulted from increments in polymer chain packing during orientation, caused a significant increase in *E_d_* values [43]. In addition, the significant increase in *E_d_* values could have occurred due to thermal fluctuations that originated from dispersion forces or long-range van der Waals [44] forces. The single oscillator model, in the case of zero photon energy (*hυ* = 0), can be utilized to obtain a polymer’s static refractive index (*n*_0_) [45];

(5) $${n_{0}}^{2}=\left(1+\frac{E_{d}}{E_{0}}\right)$$

The calculated *n*_0_ values increased from 1.43 to 4.42 when the dopant (SrTiO_3_/CNTs) mass increased from 0 wt% to 1 wt%. It is well known that polymer films which have a high refractive index are qualified to be applied in optoelectronic devices. The addition of SrTiO_3_/CNTs as a dopant increased the refractive index of the PVA polymer films, and hence increasing the amount of added dopant enhanced the optical properties of the films. Therefore, these prepared films could be employed in display screens, encapsulations of organic light-emitting diodes, image sensors, and fabrication of plastic lenses that are used in eyeglasses [46,47].

Figure 8b shows the plot of the *ɛ*_2_ as optical dielectric loss which is expressed by 2*nk* vs. *hv* for the doped PVA films with different masses of SrTiO_3_/CNTs [48]. From this figure, the interception of the extrapolated linear part of the curve along the *hυ* axis gives the values of the real band gap (E_r_), which is a crucial parameter in determining the recommended optical transitions [49].

The E_r_ values are relatively close to the values of E_ind_ along with all additions of the dopants. Due to these outcomes, the majority of optical transitions in these films follow the indirect transitions. The oscillator strength (*f*) of doped polymer films is another important parameter that affects their optical performance. There are some key factors that play major roles in tuning the oscillator strength (*f*) of the films, such as the chemical structure and the molecular mass of the polymer, film thickness, and molecules’ aggregation in the polymer film. Knowing these factors helps to produce polymer film with optimal optical properties for intended applications. Equation (5) can be used to calculate the oscillator strength of the polymer film [50];

(6) $$f=E_{d}E_{0}$$

The oscillator strength values of the PVA films are summarized in Table 2 and gradually increase with the additions of SrTiO_3_/CNTs. The molecular electronic structure depends on the chemical structure of the polymer films, and thus linear optical susceptibility (*χ*^(1)^) and third order nonlinear optical susceptibility (*χ*^(3)^) are controlled by their electronic structure. Some applications of the polymer films, i.e., optical data storage and optical switching, rely on their linear and nonlinear optical susceptibilities [51].

The *χ*^(1)^ and *χ*^(3)^ values of PVA/SrTiO_3_/CNT films summarized in Table 2 are calculated from Equation (7) [52];

(7) $$\chi^{\left(1\right)}=\frac{E_{d}/E_{0}}{4\pi },\;\;\;\;\;\;\;\;\chi^{\left(3\right)}=6.82\times 10^{-15}{\left(E_{d}/E_{0}\right)}^{4}$$

The dopants can interact with the polymer and result in the enhancement of the local electric field as well as create new energy levels, and thus increase *χ^(^*^3)^ [53]. The polarizability of the molecules is dependent on the chemical structure of the polymer molecules and therefore influences the nonlinear refractive index (*n*_2_) [54]. The *n*_2_ is a crucial parameter to determine the suitable applications for the prepared polymer films which can be calculated by Equation (8) [55,56];

(8) $$n_{2}=\frac{12\pi x^{\left(3\right)}}{n_{0}}$$

It is obvious that as we increased the content of SrTiO_3_/CNTs, the polarizability of the polymer molecules enhanced and thus increased the nonlinear refractive index (*n*_2_), as reported in Table 2. The linear and nonlinear refractive indices of polymer films are improved for the PVA films with the additions of the SrTiO_3_/CNTs. Therefore, the prepared films have high potential to be used in optoelectronic applications. Some optical parameters of PVA/SrTiO_3_/CNT polymer films, i.e., E_dir_, E_ind_, *n*_0_, *χ*^(3)^, and *n*_2_, were used for comparison with previous studies, as reported in Table 3. The value of E_dir_ in this study is relatively smaller than the values of previous studies. The values of *n*_0_, *χ*^(3)^, and *n*_2_ are greater than those reported in previous studies, which demonstrates the preference of PVA/SrTiO_3_/CNT polymer films for optoelectronic applications.

## 4. Conclusions

In conclusion, this study successfully fabricated PVA/SrTiO_3_/CNT polymer nanocomposite films using the solution casting method. The SrTiO_3_ nanoparticles exhibited a cubic crystal structure with an average crystal size of 28.75 nm. Incorporation of SrTiO_3_/CNTs up to 1 wt% resulted in an increase in the lattice parameter of SrTiO_3_ within the PVA polymer film from 3.91 Å to 3.95 Å. Additionally, the inclusion of SrTiO_3_/CNT nanofillers up to 1 wt% significantly improved the optical properties of the PVA films. Moreover, the thermal stability of the films was enhanced, with the onset of the second degradation stage increasing from 235 °C for pure PVA to 310 °C for doped PVA. SEM images showed a uniform distribution of 0.3 wt% SrTiO_3_/CNTs in the PVA polymer films, while some agglomerations were observed in films containing 0.7 and 1.0 wt% SrTiO_3_/CNTs. The direct and indirect optical band gaps of PVA films decreased with increasing mass percentage of SrTiO_3_/CNTs. Furthermore, the single-oscillator energy (*E*_0_), dispersion energy (*E_d_*), and optical susceptibility values increased with increasing nanofiller weight, with calculated *n*_0_ values ranging from 1.43 to 4.42. Additionally, improvements in nonlinear refractive index values were observed due to the inclusion of SrTiO_3_/CNTs. These findings suggest that the fabricated films exhibit promising optical and thermal properties suitable for optoelectronic applications. Future research efforts will focus on investigating the electrical properties of PVA/SrTiO_3_/CNT polymer nanocomposite films through electrical measurements.

## Figures and Tables

**Table 1:** Optical parameters of SrTiO_3_/CNT doped PVA films.

SrTiO_3_/CNT	E_ind_ (eV)	E_dir_ (eV)	*E*_0_ (eV)	*E_d_* (eV)	*n*_0_
0.0	5.31	5.06	4.48	4.69	1.43
0.3	5.10	4.86	5.12	20.81	2.25
0.7	5.08	4.76	4.84	54.11	3.49
1.0	4.92	4.50	4.98	92.57	4.42

**Table 2:** Summarized dispersion parameters of PVA/SrTiO_3_/CNT polymer films.

SrTiO_3_/CNT (wt%)	*f* (eV^2^)	*χ*^(1)^ (esu)	*χ*^(3)^ *x* 10^−13^ (esu)	*n*_2_ *x* 10^−12^ (esu)	E_r_ (eV)
0.0	21.01	0.086	0.097	0.25	5.30
0.3	106.54	0.32	18.61	31.18	5.01
0.7	261.9	0.89	1065.40	1150.85	5.12
1.0	460.99	1.48	8142.32	6944.75	5.05

**Table 3:** The optical parameters, i.e., E_dir_, E_ind_, *n*_0_, *χ*^(3)^, and *n*_2_, of PVA/SrTiO_3_/CNT polymer films compared with optical parameters of other related polymer nanocomposites.

SrTiO_3_/CNT (wt%)	E_ind_ (eV)	E_dir_ (eV)	*n*_0_	*χ*^(3)^ *x* 10^−13^ (esu)	*n*_2_ *x* 10^−12^ (esu)	Ref.
PVA + 1% Fe_2_O_3_ NPs	4.8	------	1.62	0.46	1.06	[57]
PVA/PVP/PB-Nd^+3^	4.82	5.83	------	8.478	15.33	[5]
PVA − 3%ZnO	4.89	5.45	1.58	------	------	[58]
PVA + 1% NiO NPs	5	------	1.87	2.66	5.35	[57]
PVA-Y_2_O_3_	4.54	5.31	2.77	134.43	183	[59]
PVC/PVP/0.7%SrTiO_3_	5.8	5.96	3.20	491.75	580.04	[3]
PVDF/SrTiO_3_/0.7%CNTs	5.30	5.53	4.27	5977	5280.9	[17]
PVA/SrTiO_3_/CNT	4.92	4.50	4.42	8142.32	6944.75	This work

## Data Availability

Data are contained within the article.

## Supplementary Materials

The following supporting information can be downloaded at: https://www.mdpi.com/article/10.3390/polym16101392/s1. Figure S1: EDX elemental mapping analysis of the prepared samples (a) PVA, (b) PVA/SrTiO_3_/CNTs, (3wt%) (c) PVA/SrTiO_3_/CNTs (7wt%), and PVA/SrTiO_3_/CNTs(10wt%).
