# Supplementary material for: Structural, Optical, and Thermal Properties of PVA/SrTiO3/CNT Polymer Nanocomposites
Source: Polymers (Basel). 2024 May 14;16(10):1392. doi: 10.3390/polym16101392 (PMC11124778; doi:10.3390/polym16101392)
Supplement: Supplementary file 1 [file polymers-16-01392-s001.zip › polymers-2912049-supplementary.pdf]

Supporting Information

Structural, Optical, and Thermal Properties of PVA/$SrTiO_3$/CNT Polymer Nanocomposites

Alhulw H. Alshammari

Physics Department, College of Science, Jouf University, Sakaka P.O. Box 2014, Saudi Arabia; ahalshammari@ju.edu.sa

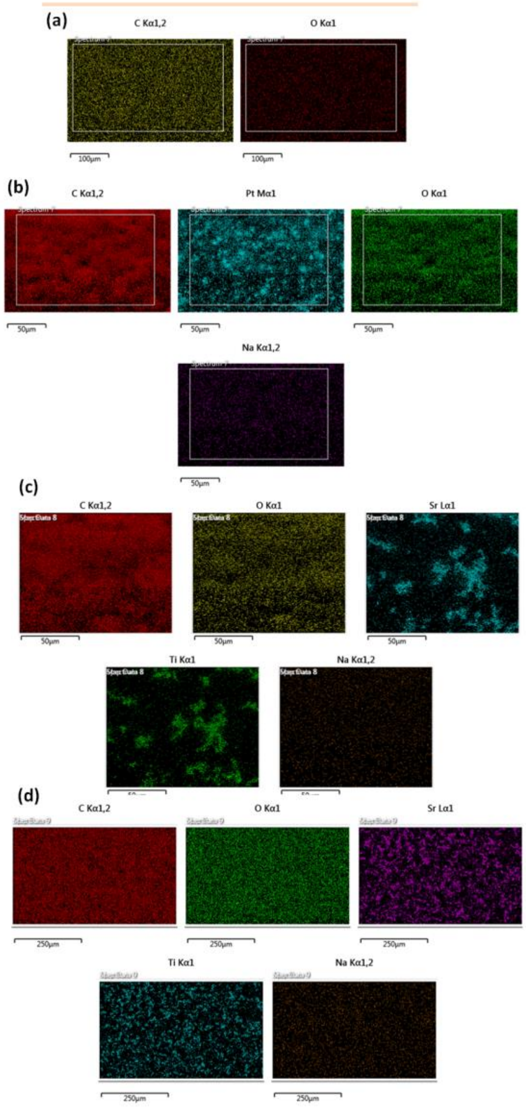

Figure S1. EDX elemental mapping analysis of the prepared samples (**a**) PVA, (**b**) PVA/$SrTiO_3$/CNTs, (3wt%) (**c**) PVA/$SrTiO_3$/CNTs (7wt%), and (**d**) PVA/$SrTiO_3$/CNTs (10wt%).
